# Novel Tactile Sensor Technology and Smart Tactile Sensing Systems: A Review

**DOI:** 10.3390/s17112653

**Published:** 2017-11-17

**Authors:** Liang Zou, Chang Ge, Z. Jane Wang, Edmond Cretu, Xiaoou Li

**Affiliations:** 1 Department of Electrical and Computer Engineering, University of British Columbia, Vancouver, BC V6T 1Z4, Canada; cge@ece.ubc.ca (C.G.); zjanew@ece.ubc.ca (Z.J.W.); edmondc@ece.ubc.ca (E.C.); 2 College of Medical Instruments, Shanghai University of Medicine and Health Sciences, Shanghai 201318, China

**Keywords:** smart tactile sensing, microfabrication, machine learning, sensor fusion

## Abstract

During the last decades, smart tactile sensing systems based on different sensing techniques have been developed due to their high potential in industry and biomedical engineering. However, smart tactile sensing technologies and systems are still in their infancy, as many technological and system issues remain unresolved and require strong interdisciplinary efforts to address them. This paper provides an overview of smart tactile sensing systems, with a focus on signal processing technologies used to interpret the measured information from tactile sensors and/or sensors for other sensory modalities. The tactile sensing transduction and principles, fabrication and structures are also discussed with their merits and demerits. Finally, the challenges that tactile sensing technology needs to overcome are highlighted.

## 1. Introduction

Benefiting from the sense of touch, we learn to delicately perceive, grasp and manipulate a wide range of objects. It is an important way to sense and interact with the world. Recent years has seen increased exploration of tactile sensing [1]. Given the importance of tactile sensing in daily life and industry, researchers have been striving to understand this sense, and aim to develop smart tactile sensing systems which can facilitate people’s life [2,3,4,5]. Tactile sensors range from simple sensors for sensing the location of contact to more complex sensors used to measure surface properties, such as roughness, stiffness and temperature. There are innumerable applications of tactile sensing systems of which most people are never aware, such as manual palpation and prosthetic limb.

The research on smart tactile sensing has attracted intense research interest in different fields because of its diverse applications, from industry to biomedical engineering. Great efforts have been made to develop advanced tactile sensors using new transduction techniques and materials [2,6]. Over the past two decades, a wide variety of tactile sensors able to acquire various contact parameters have been reported in the literature, exploring almost all possible modes of transduction [2,6,7,8,9,10,11,12,13]. For instance, Drimus et al. developed a novel tactile sensor based on piezoresistive rubber and thread electrodes [8]. They further utilized these sensors for classification of rigid and deformable objects. In [9], a flexible and stretchable durable fabric-based tactile sensor capable of capturing typical human interaction forces was developed. There are numerous tactile sensors able to acquire more than one type of characters of the object to be contacted. A highly sensitive tactile sensor using free-standing Zinc Oxide or Polyvinylidene Difluoride (ZnO/PVDF) thin film with graphene electrodes is developed for monitoring pressure and temperature simultaneously [10]. However, the usage of tactile sensor in practical applications is still limited [4]. One of the main reasons stems from the difficulties with the processing of acquired data from tactile sensors [4,14]. Compared to other senses, such as visual and hearing, the properties of tactile sensor data are much more variable [15]. The signals from tactile sensors can be noisy, high-dimensional, complex and contain irrelevant information as well as essential one [16]. There still is lack of signal processing and machine learning methods that can deal with such complicated problems [16].

An effective tactile sensing system should be endowed both advanced tactile sensors and/or sensors for other sensory modalities, which are able to perceive information from the environment, and intelligent signal processing tools capable of interpreting the measured information and making decisions [17,18]. However, to our knowledge, there is no smart sensing system capable of perceiving and interpreting surrounding information at the same level as the human somatosensory system yet [19]. In addition, the penetration of tactile sensors in industrial and biomedical applications is still extremely low [4]. Most of the existing smart tactile systems are still mainly research tools.

The development of smart tactile sensing systems is still an open problem with many technical and scientific challenges. It requires strong interdisciplinary efforts, not only for advanced tactile sensors, but also for appropriate algorithms to deal with the acquired data. Smart tactile sensing can be enhanced by advances in the utilized materials, fabrication technologies and signal processing. Although some topics tackled in this paper may overlap with some previous surveys, the focus of this review is different. This paper extends previous reviews by focusing on the current state-of-the-art machine learning and signal processing technology, outstanding challenges which must be overcome, and applications of smart tactile sensing technology. In addition, novel physical principles, material processing methods, and more recent developed fabrication technologies that can contribute to the hardware development of tactile sensing systems are also discussed.

## 2. Tactile Sensing Principles and Structures

Tactile sensing principles refer to the mechanisms coupling the non-electrical domain with the electrical domain. Efficient domain coupling principles can be generally divided into four main types: capacitive, piezo-resistive, piezoelectric and optic. The four principles are preferred due to their more robust implementation of functional structures at the Microelectromechanical Systems or Nanoelectromechanical Systems (MEMS/NEMS) level.

### 2.1. Capacitive Tactile Sensors

The philosophy behind capacitive tactile sensing is changing the capacitance by mechanically changing the geometry of a capacitor. The capacitance of a parallel capacitor, the classic capacitor structure used for capacitive sensing, can be calculated as:

(1) $$C=\frac{\varepsilon wl}{d}$$

In Equation (1), *ε* is the di-electrical permittivity, w is the width of the overlapped area, *l* is the length of the overlapped area of the two parallel plates, and *d* is the gap between the two parallel plates. Capacitive sensors generally have good frequency response, high spatial resolution and large dynamic ranges [2], though they might be susceptible to multiple types of noises [2].

Parallel plate capacitors are the fundamental structure for capacitive sensing. Sensing plates have at least one degree of freedom for geometry variance to change capacitance [2,20]. Mesa structures are generally used on the movable plate to promote the contact with the sensing target [11,21,22,23,24,25,26,27,28,29]. Rectangular stripes [21,29], pyramidic structures [11,22,24,25,26,27], spheres [26], and pillars [11,27] have all been used in similar applications. As for readout circuit, current or voltage setup can be used to sense the capacitance change by the transient voltage or current output changes respectively [22]. RCL oscillation circuit can be used to sense the change of capacitance by the shift of resonant frequencies [23]. To the best of the authors’ knowledge, the best performance of capacitive tactile sensors ever reported is a minimum resolution of 3 Pa with a sensitivity of 0.55 kPa^−1^ [27].

### 2.2. Piezo-Resistive Tactile Sensors

Piezo-resistive tactile sensing is implemented by mechanically changing resistivity of a sensing structure. Piezo-resistive effect for a conductive (semi-conductive) material can be described as [30]:

(2) $$\frac{\Delta R}{R}=\left(1+2\sigma +\pi E\right)\chi$$

(3) $$R=\rho \frac{L}{A}$$

(4) $$\pi =\frac{\Delta \rho }{\rho }T$$

(5) $$E=\frac{T}{\chi }$$

In Equation (2), *R* is the electrical resistance of a conductive structure along longitudinal direction, and ΔR is the corresponding change of resistance as a result of applied strain. *σ* is the Poisson’s ratio of the material. *π* is the piezo-resistive coefficient. *E* is the Young’s modulus. *χ* is the longitudinal strain. Equation (3) is the classic calculation of electrical resistance. Equation (4) is the math computation for piezo-resistive coefficient along one crystal orientation. Equation (5) describes the relationship between stress and strain. Piezo-resistive sensing is robust against noise, making it a better choice for array based applications [2,20]. As for limitations, piezo-resistive sensing is heavily affected by hysteresis, leading to a lower frequency response, and it can only be used for dynamic measurements with limited spatial resolution [2].

Effective piezo-resistive sensing can be implemented as long as the sensor can efficiently contact the sensing object. Diaphragms or cantilevers are sometimes used to increase sensing efficiency by increasing mechanical deflection and stress [31,32,33,34]. For a piezo-resistive sensing system, the read out circuit can be as simple as a DC biased Weston bridge. The reported maximum piezo-resistive sensing sensitivity can reach 0.25 mV/nm [33].

### 2.3. Piezoelectric Tactile Sensors

There are two different sensing principles for piezoelectric tactile sensors: passive and active. Passive tactile sensing takes advantage of direct piezoelectric effect. As a result of the material polarization under external stress, electrical charge is generated. The generated electrical charge density can be expressed as [35]:

(6) $$D_{i}=d_{ijk}\chi_{jk}$$

In Equation (6), *D_i_* is the generated charge density at *i* orientation in a Cartesian coordinate system, and *d_ijk_* is the direct piezoelectric coefficient of the material. *χ_jk_* is the external stress signal as sensing input. Active tactile sensing takes advantage of converse piezoelectric effect. The piezoelectric sensing structures are electrically actuated under its first-order resonant frequency. When an external stress is applied, a resonant frequency shift linear to the external stress is generated. The resonant frequency can be calculated as [36]:

(7) $$f_{0}=\frac{1}{2Z}\sqrt{\frac{K}{\rho }}$$

*Z* is the thickness of the piezoelectric materials. *K* is the stiffness constant of the material, and *ρ* is the density of the material. The resonant frequency shift under external stress can be considered directly linear to the external stress [36]. Piezoelectric tactile sensors exhibits very high frequency response, making them the best choice for dynamic signal sensing [2].

The sensing principle leads to sandwich structures as the general sensing structures for piezoelectric tactile sensors. Piezoelectric layers are deposited between two electrode layers. Similar to the capacitive tactile sensors, convex structures like mesas, spheres have been integrated as a contact promoter [37,38]. For piezoelectric tactile sensors, electrical readout circuit presents either voltage or current changes as a result of the external stress, or frequency shift. The maximum measurable pressure range has been reported as 100 MPa [39].

### 2.4. Optical Tactile Sensors

Optical tactile sensing is implemented by coupling geometric change of electromagnetic waveguide with the modulation of wavelength, phase, polarization or intensity of the wave [40]. Optical tactile sensing is immune to electronic noise [41]. Optical sensors generally have high spatial resolution and wide dynamic response range [2]. Optical tactile sensing can be used for sensing surface roughness [42], compliance [40], shear and vertical stress [43,44,45]. For roughness measurements, the reported best resolution is around ~100 nm level [42]. Key sensing structures for tactile roughness sensing are optic fibers vertically placed towards the sensing surface [40,42]. Waveguides sandwiched between substrate and contact interface structures are commonly used for tactile stress sensing [43,44,45,46]. For stress and mechanical forces, a resolution of 0.02 N has been reported for optical sensors used for the minimally invasive surgery [47]. Optical tactile sensing has shown great potential in applications requiring flexibility and portability [48,49]. Optical fibers are able to cooperate with other sensing principles to significantly improve the system performance and enhance the robustness to electromagnetic disturbance [49]. In addition, optical fibers have been used for both contact tactile sensing and proximity tactile sensing. 3D printed, deflectable mini structures are used to couple the mechanical signal with the optical signal through a reflective surface. It can significantly enhance the grasping capability of a robotic arm [48]. In conclusion, optical tactile sensors can provide high sensitivity [40,41,44,45,47,50].

### 2.5. Trade-offs and Challenge

In this section, the four basic tactile sensing principles and the electrical interfaces are introduced. As to other sensing principles such as strain gauges [2,20], they take advantage of at least one of these four principles to couple the external sensing signals into readout electrical signals. A comparison between different sensing principles is provided in Table 1.

In this review, the capability of integration is emphasized because it is vital to multi-domain tactile sensing. The capability of integration is discussed from two perspectives: integration with electrical interface; and integration of functional sensing structure for multi-domain signals. For all of these four sensing principles, research has demonstrated their capabilities of integration in both perspectives.

For the integration with electrical interfaces, system level integration (printed circuit board level) has been extensively studied, verified and applied for all of these four principles [11,24,25,26,27,28,29,31,38,40,41,47,48,49,50,51,52,53,54,55]. Integrating functional sensing structures into a single micro/nanodevice is a more recent research trend, and significant progress has been reported. Capacitive tactile sensors have been fabricated by post-Complementary Metal Oxide Semiconductor (post-CMOS) fabrication technologies on Metal Oxide Semiconductor Field Effect Transistors (MOSFETs) [23]. Piezo-resistive tactile sensors can be fabricated by similar processes [32,56]. Dahiya et al. developed new tactile sensing chips consisting of array of Piezoelectric Oxide Semiconductor Field Effect Transistors (POSFETs) tactile sensing devices [57]. Electrical charge accumulates under external force. Polymer optical tactile sensors have been fabricated by photoresist as waveguide with photovoltaic microstructures [58]. Micro Optical Electrical Mechanical System (MOEMS) have also been fabricated for tactile sensing applications [59,60,61,62,63,64]. Quantum dots can be used as solid state light source and electrical interface for optical tactile sensing at the level of micro/nanodevices [58,59,60,62].

For sensing structure integration, capacitive sensing structures with piezo-resistive layers have enabled the measurement of dynamic properties such as acceleration and displacement (including thermal induced displacement) as well as hardness and contact forces [34]. In addition, integration of multiple MEMS sensors into a single Printed circuit board (PCB) level chip has been proved feasible for multiple domain tactile sensing [65]. Integration of MEMS transducers on micro optic fibers enable multiple domain tactile sensing for DaVinci robots [66]. Optical tactile sensing has a unique advantage in sensing structure integration. Optical tactile sensors, especially fiber based ones, can be used for multi-domain sensing with a single type of structures [48]. For instance, hard and soft mechanical sensing, as well as distance sensing can all be implemented through the measurement of reflected laser intensity [48].

## 3. Materials for Tactile Sensing

### 3.1. Capacitive Tactile Sensors

Capacitive sensing structures can be fabricated by various types of materials. Besides the possibilities of forming sandwich, parallel capacitors, good mechanical properties are required. Polysilicon has been one of the major material types for capacitive tactile sensor [21,22,23,28]. Polymer materials, typically Polydimethylsiloxane (PDMS) [24,26,27] and SU-8 [67], have become more and more popular. These polymer materials have acceptable chemical stability and elastic properties. Polymer materials open the field of flexible tactile sensor devices [24,25,26,27]. In a flexible capacitive sensor, polymer materials can be used for di-electrical intermediate layers [27], movable sensing plates, or the 3D contact promoters [11,24,25,26].

### 3.2. Piezo-Resistive Tactile Sensors

Electrical resistivity and mechanical elasticity are necessary for piezo-resistive sensing. These are common physical properties for most metal, semiconductor, and some polymer materials, which are therefore candidates for piezo-resistive sensing structure. In early years, piezo-electrical tactile sensors have been fabricated in single crystal silicon and poly-silicon [31,32,33,34]. Recently, research works have extensively demonstrated the piezo-resistivity in carbon nanomaterials such as multi-walled carbon nanotube and graphene related materials [53,54,68]. Carbon black micro/nanoparticles have also been shown to be piezo-resistive [55]. When used in the fabrication of piezo-resistive tactile sensors, additional supportive polymers are usually used for carbon materials. Typical examples include polystyrenes [55], poly-urethanes [54,68], and PDMS [53].

### 3.3. Piezoelectric Tactile Sensors

The dependence of piezo-electrical property narrows available options of material for piezoelectric tactile sensors. For rigid sensors, quartz [35], zinc oxide [35,39,69] and lead zirconated titanate (PZT) [36,52,70,71,72] are popular materials. Piezo-electrical sensing structures for flexible tactile sensors have been fabricated based on zinc oxide nanomaterials [39,69]. Polyvinylidene fluoride (PVDF) is a more popular choice for flexible piezoelectric tactile sensors [37,38,57,70,73,74,75,76]. More recent research about piezoelectricity in cellulose materials indicates a novel material for flexible piezoelectric tactile sensor fabrication [77,78].

### 3.4. Optical Tactile Sensors

Optic waveguide materials are the key material for optical tactile sensors. Dating back to 1970s, composite like ZnCl_2_ glass has been proposed as fiber materials [79]. Silica materials are very good options for single-mode optical fibers [80]. Conventional polymer fibers include poly(methyl-methacrylate) (PMMA), polystyrene(PS), polycarbonate(PC), polyurethane(PU) and epoxies [81]. Newly explored optical polymers includes: deuterated and halogenated polyacrylates, fluorinated polyimides, benzocyclobutene, perfluorovinyl ether copolymers [81]. Materials for supportive structures include acrylic polymers [43], PDMS [44] and nitinol [41]. Materials for optical tactile sensors have to fulfill certain optical transparency and elasticity requirements.

### 3.5. Material Functionalization: Towards Multiple Domain Tactile Sensing

A recent trend in tactile sensor material development is material functionalization in order to implement certain properties for certain tactile sensing principles. Material functionalization greatly expands the potential material options for tactile sensing structures.

Physical functionalization includes surface functionalization and bulk functionalization. The typical bulk functionalization method is mixing additives into the target materials for certain functional properties. Once the concentration of the functional additives reaches a threshold, known as the percolation threshold, the mixture starts to exhibit the corresponding functional properties for certain applications. Silver nanoparticles can be mixed into SU-8 photoresist, which is originally an electrical insulator, to fabricate suspended membrane structures suitable for both capacitance tactile sensing and piezo-resistive tactile sensing [67]. Carbon black micro particles have been mixed into thermal plastic for piezo-resistive sensors [55]. Cellulose nanocrystals have also been mixed into photo-curable matrix for piezoelectric applications [77,78]. Surface functionalization is 2D or 3D functional coating on the non-functional surface. The key factor for consideration is the adhesion. Conductive layers can be coated on the polymer suspended structure surface to get capacitive or piezo-resistive sensors [24,25,26,27,54,67,68]. In a sequence of piezoelectric functional coating followed by conductive functional coating, flexible piezoelectric tactile sensors can be fabricated [76].

Chemical functionalization can be done by bonding functional groups for specific sensing purposes to large molecules. The bonded functional groups enable additional sensing capability of the material. Multi-walled carbon nanotubes (MWNTs) have always been ideal subject to chemical functionalization [82,83,84,85]. After chemical functionalization, carbon nanotubes can also be used for thermal tactile sensing [86] or sensing for specific chemicals [85,87,88] besides their traditional piezo-resistive applications [54], making them ideal candidates for artificial skin [88,89,90]. 

### 3.6. Comparisons and Trade-Off Discussion

Advantages and disadvantages of different material types are listed in Table 2. Table 2 focuses on structural materials for tactile sensors. For nanomaterials such as graphene, carbon nanotubes and nanowires, they have to reply on at least one of the structural materials for the sensing purposes.

## 4. Fabrication Technology

### 4.1. Standard Fabrication: Micromachining and Molding

**Figure 1:**
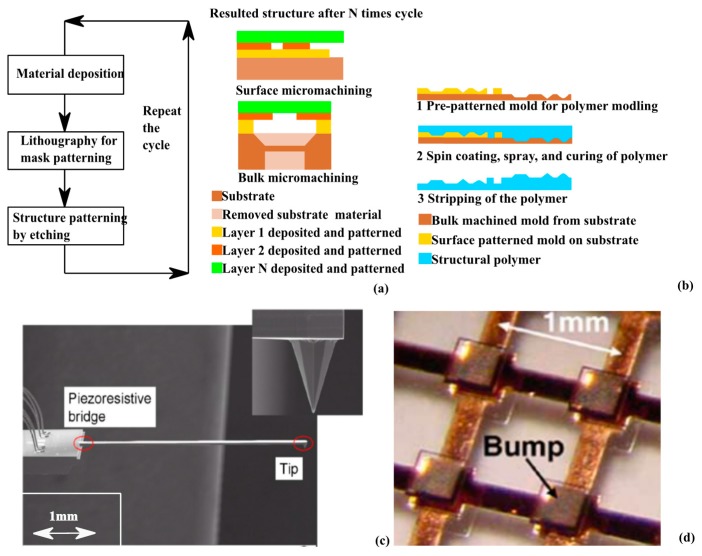
Conventional tactile sensor fabrication technologies: (**a**) Micromachining, (**b**) Molding; and (**c**) (Reprinted with permission [33]. Copyright © 2014, Springer Berlin Heidelberg) and (**d**) (Reprinted with permission [24]. Copyright 2006, IEEE) examples of micromachined and modeled tactile sensing structures.

Micromachining is the most important fabrication technology for tactile sensors. Tactile sensors can be directly fabricated by micromachining [21,22,23,28,31,32,34,71], or vital fabrication pre-requisites are fulfilled through micromachining process, such as molds for polymers [24,26,27]. The fundamental flow of micromachining is illustrated in Figure 1a.

As illustrated in Figure 1a, micromachining process can be summarized as material deposition-masking-patterning cycles. This cycle is necessary for not only structural materials, but also sacrificial materials. For instance, photoresist may not withstand dry etching or wet etching of certain material, so additional cycles in Figure 1a is required to first pattern layers of masking material. The total repeat times of a single micromachining cycle are determined by the complexity of the structures to fabricate and the structural material properties. Micromachining can be subcategorized into surface micromachining and bulk micromachining. In surface micromachining, processed materials are stacked layer by layer on substrates. No process is done on the substrate material, while substrate material is also structured in bulk micromachining. Surface micromachining is generally used to fabricate tactile sensors integrated with IC level electrical read out circuits [23,34,57]. Bulk micromachined tactile sensors are generally standalone, and additional bonding steps are required to interface electrical read out circuits. Depending on the material properties and compatibility with etchant, the order of process for different materials have to be carefully considered to guarantee patterned layers to survive the process followed. 

Molding/imprinting is the second popular and important fabrication method for tactile sensors. It is mainly used for polymer based tactile sensors for low cost or flexible applications. Molding fabrication can be done at both the macroscale and the microscale; nevertheless, lithography steps or micromachinings are always required to first pattern inversed structures in order to successfully mold tactile sensing structures. A typical molding process flow is illustrated in Figure 1b. To fabricate complex 3D tactile sensing structures, molded polymers have to be bonded through adhesive bonding or oxygen plasma bonding [24,26,27].

### 4.2. Lithography Based Rapid Micro 3D Fabrication

Some recently developed 3D fabrication technologies can greatly simplify the fabrication process for tactile sensors. A typical example is the lithography based polymer MEMS fabrication technology [67,91,92,93,94,95]. Using cross-linkable epoxy negative permanent photoresist, high resolution and rapid micro-3D fabrications can be implemented by tuning the exposure dose emitted towards the photoresist to partially trigger post exposure cross-linking. This technology is suitable for the fabrication of capacitive and piezo-resistive tactile sensors, and it also has the potential of fabricating support structures for micro optic tactile sensors.

### 4.3. Comparison of Fabrication Technologies

When choosing a fabrication technology for specific tactile sensors, process cost, complexity, robustness are the major factors for consideration. For the four principles discussed, the advantages and disadvantages of major fabrication technologies are compared in Table 3.

Many capacitive tactile sensors require suspended, movable 3D structures for efficient sensing. The fabrication of this type of structures is generally more complicated than fixed structures, and the releasing process suffers from the stiction problem: due to the surface tension after releasing by wet etching, sometimes the fabricated structures will collapse to the substrate. In some cases of piezo-resistive sensors [31,32,34], suspended 3D structures are used to promote deformation. This type of piezo-resistive sensors suffers from the similar stiction problem. For piezoelectric and optical sensing, only fixed structures are required. This makes the micromachining based fabrication much simpler. However, optical tactile sensors are generally stricter on defects and geometrical variation of deposited layers, slightly reducing the robustness. Molding and imprinting are generally simpler than micromachining, but the fabrication of molds relies either on micromachining or 3D fabrications on a substrate, leading to additional process steps and complexity. For suspended 3D structures, the bonding process of molded structures is done in gaseous or vacuum ambient, eliminating the stiction problem. Rapid 3D fabrication methods are simpler than molding since the polymer structures can be directly patterned based on the design. For both molding and 3D fabrication, suitable materials are neither electrically conductive nor piezoelectric. Additional surface processes are generally required to make the devices functional. Most polymers have satisfying optical properties. Therefore, molding and rapid 3D fabrications are good choices for fabrication of optic tactile sensors.

## 5. Smart Tactile Sensor Applications

Compared with other perception methods, e.g., visual or hearing, tactile sensing was relatively neglected in the early years of robotics. Both research and industry communities have been directing their attentions toward tactile sensing technologies since 2000 [2]. Tactile sensors have diverse applications in different fields, which have been reviewed and reported in the literature [2,7,18,96]. Great efforts have been made to develop high-performance tactile sensors. In the last decade, tactile sensing has attracted increasing interest mainly due to its applications in three domains, including artificial skin for robotics, tactile sensing for unstructured environments and biomedical applications [3,4,18,97,98,99,100]. In this section, we will discuss state-of-the-art applications related to these three domains and present some research challenges.

### 5.1. Tactile Sensing for Artificial Skin (E-Skin)

The sense of touch enables tactile sensors to assess physical properties of the object, such as force, temperature, size, hardness and texture, and allow it to detect slip and control parameters in manipulation. Inspired by the human skin, significant progress in the development of artificial skin based on tactile sensing has been achieved in recent years and a wide variety of tactile sensors capable of mimicking the human skin have been reported in the literature, such as wearable devices and smart robots [4,101,102,103,104,105]. Artificial skin requires the macroscale integration of a large number of single sensing elements on a thin flexible substrate [104]. This cannot be achieved by simply aggregating them. Inorganic crystalline semiconductors show an advantage as they can provide high carrier mobility with excellent mechanical flexibility. Takei et al. integrated parallel semiconductors nanowires as the active-matrix backplane of a flexible pressure-sensor array [104]. Harada et al. developed a fully printed fingerprint-like three-axis tactile force and temperature sensor for artificial skin applications. The proposed artificial skin device utilized a fingerprint-like structure which allows tactile and slip force to be detected as the human skin. The authors printed multifunctional sensors on a flexible substrate, rather than using semiconductor infrastructures. It can significantly reduce the fabrication cost and enhance the feasibility of tactile sensors in commercial applications [103]. To ensure energy autonomy, recently, Núñez et al. developed a transparent tactile e-skin along with single layer graphene and coplanar interdigitated capacitive electrodes [106]. They also demonstrate the feasibility of large-scale and low-cost fabrication of a flexible and transparent e-skin for pressure sensing on a prosthetic hand [106]. Table 4 lists some existing tactile sensors developed for artificial skin.

Significant progress in the development of sensing material and sensing technology have been achieved [23]. However, the function of artificial skin always was hindered by limitations in microfabrication and cost issues [45], and only a few approaches have been successfully employed in practical robots [45]. In addition, the majorities of the researches in the literature focused on the development of fabrication of sensors which are sensitive to a specific property of the object [45]. To mimic human skin, the need for developing a sensor or a sensing system that can provide more types of properties is increasing.

### 5.2. Tactile Sensing for Unstructured Environments

Even some attempts have been made recently to mimic human skin and sense of touch [2,3,4,110], tactile sensing has rarely been used in complex robotic applications, especially in unstructured environments [18]. Unlike traditional industrial robots, such as manipulator arms, which follow a predefined and simple program, smart robots are designed to work autonomously and interact with the surrounding environment [18]. It requires that the smart robot can feel and interpret the environment with the help of various sensors [18]. Tactile sensing is crucial for safe interactions of robots and the surrounding environments, because it provides the most direct haptic feedback to control the force during the interactions [96]. It was shown that the remote tactile sensing is preferable in many unstructured environments where other sensing modality, such as vision or hearing, are limited [111]. In [112], the authors analyzed the reason why robots are glorious in factory while incompetent at the home. Outside of controlled environments, it is difficult for robots to perform sophisticated tasks without the operation command from a human. 

To interact with the environment and autonomously learn, fusion-of full-body tactile sensors and other types of sensors is shown to be indispensable [6]. Researchers from Stanford University incorporated actuated smart staffs with vision and tactile sensing, and developed a smart platform SupraPed [113]. Simulation results demonstrated that the proposed control framework significantly enhanced the locomotion performance of humanoid robots in unstructured environments. Inspired by the actuation and sensing in biological systems, Jain et al. enabled the robots to manipulate effectively with haptic sensing in unstructured environments [114]. They presented the potentiality of data-driven machine intelligence methods to inform robot about the forces that they probably encounter when performing specific tasks. The growth of robotic applications in unstructured environments has created a pressing need for smart tactile sensing systems with advanced tactile materials and fabrication technologies.

### 5.3. Tactile Sensing for Biomedical Applications

**Figure 2:**
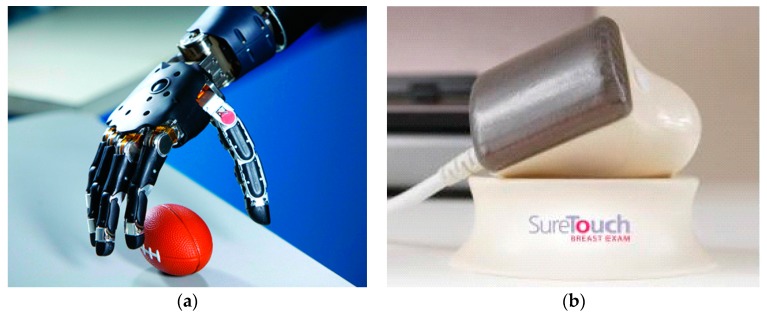
Two examples of tactile sensing systems for biomedical engineering: (**a**) the modular prosthetic limb; and (**b**) the SureTouch sensor for breast exam [120,121] (Reprinted with permission. Copyright 2015 IEEE).

During the last decade, tactile sensing has been rapidly growing, particularly in the area of biomedical engineering. The use of tactile sensing in biomedical systems has resulted in cutting edge outcomes, as reviewed by [2,97,115,116,117,118]. Here, from the standpoint of smart properties, we mainly introduce two most exciting and state-of-the-art applications in this field, including prosthesis and pattern recognition based on tactile sensing [115,119]. For more general applications, readers are referred to [2,19,97]. Based on advanced tactile sensing technologies as well as signal processing methods, researchers are able to make prosthetics that are remarkably useful and realistic [97,120]. The absence of tactile information impedes the functionality and efficiency of traditional prostheses, such as the simple peg leg. The tactile feedback from tactile sensors is essential to the amputees or people with impaired tactile sensibility. However, only a few existing prosthesis can provide effective tactile sensation feedback to users, which is mainly due to technical difficulties and the complicated nature of the human tactile system. One of the most fantastic examples is the modular prosthetic limb developed by researchers from Johns Hopkins University, as shown in Figure 2a [120]. Based on more than 100 sensors, the anthropomorphic prosthetic hand can provide high-resolution tactile and position sensing capabilities. In addition, to restore the sense of touch to the people with tactile sensation difficulties, advanced machine learning algorithms are necessary to map tactile sensor information with object properties.

The area of tactile pattern recognition has also attracted increasing attentions from researchers in signal processing and computer science. Pattern recognition methods, which will be further discussed in the next section, have been shown to be effective for interpreting information from tactile sensors in challenging applications, such as objective diagnostic palpation, cancer detection, gait analysis et al. [2,18,19]. Clinically, the doctors always use the hand and palm to evaluate the condition of organs and tissues. The main reason is that the mechanical properties of healthy soft tissues differ from the cancerous ones [19,122]. It was demonstrated that SureTouch, as shown in Figure 2b, a tactile sensing device by Medical Tactile Inc., Los Angeles, CA, USA, can provide up to four times more sensitivity than the human hand in breast cancer detection. Equipped with 192 high resolution pressure sensors, the device can detect lumps or masses as small as 5 mm, which is more sensitive than the human sense of touch. Considering the present limitations of kidney stone removal laparoscopy, Afshari et al. developed a novel tactile sensory system capable of detecting the exact location of kidney stones during laparoscopy [123] based on force sensors. This new tactile sensing system can also be applied in different fields of artificial palpation, such as detection of breast cancer and estimation of different properties of cancerous tumors [124].

## 6. Intelligent Signal Processing for Smart Tactile Sensing

**Figure 3:**
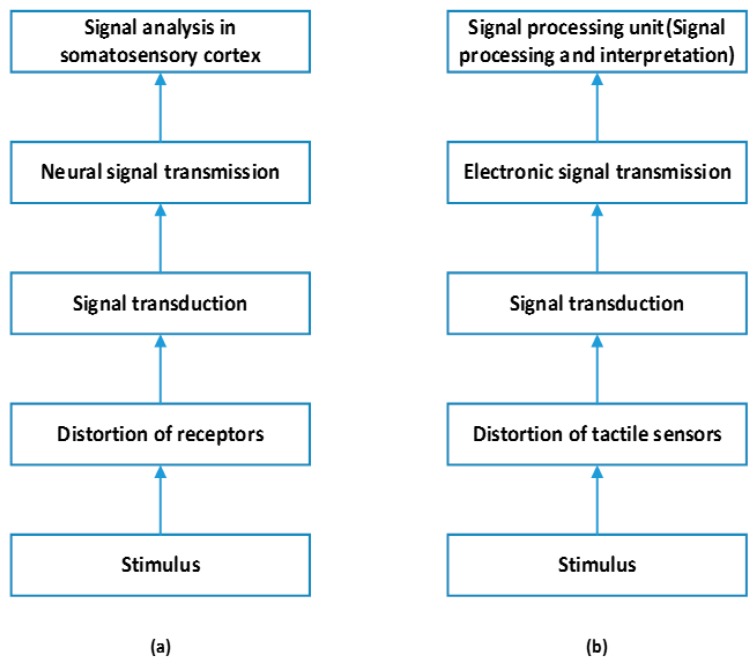
The illustrative hierarchical transmission of tactile signals in: (**a**) human skin; and (**b**) smart tactile sensing system.

Figure 3 illustrates the tactile signal transmission in the human skin. Sensory units, e.g., mechanoreceptors, distributed in human skin detect mechanical stimulations, e.g., force and texture. Then, a sequence of neural pulses is generated and transmitted to the central nervous system for further processing [97]. Similar to the human skin, the general hierarchical transmission of tactile signals in a smart tactile sensing system includes signal transduction, signal conditioning, data transmission, signal processing and control system [3,4,125]. Smart Tactile sensing is not only hardware demanding, e.g., high sensitive tactile sensors, but also much more demanding in terms of software corresponding to sophisticated neural signal processing in the brain (e.g., signal processing and data fusion techniques). In this section, we mainly discuss state-of-the-art methods which have been proposed to address the following data processing challenges: data acquisition and artifacts removal, pattern recognition and tactile sensor fusion.

### 6.1. Data Acquisition and Artifacts Removal

The first stage of tactile sensing is data acquisition, which aims to collect physical properties of the object or the contact via tactile sensors. As mentioned earlier, tactile sensors can be used to measure different types of tactile signals, such as force, temperature, size, hardness and texture. With different tasks, various types of tactile sensors can be utilized, such as sensors to measure pressure [126] and hardness [127]. The change of the signal of interest can be reflected by the change of the sensors’ electrical properties. Many tactile sensing systems also involve integrated amplifiers for the reason that the amplitude of the acquired signal is too tiny. An analog-to-digital converter digitizes the signal before further analysis. 

In addition, the research also needs to deal with challenges related to data acquisition, such as trade-off between the spatial resolution and temporal resolution, simultaneous sensing of multiple stimuli, etc. [128,129,130,131]. The massive amount of tactile signals need to be acquired at high rates. However, the increase of the number of tactile sensors limits the speed to collect the data. To provide fast sampling rate with high spatial resolution, Lee et al. took advantage of parallel processing properties of Field Programmable Gate Array (FPGA) and developed a tactile sensing system with high spatiotemporal resolution [128]. Based on the theory of compressed sensing, Hollis et al. proposed a novel strategy to reduce hardware complexity and addressed the scalability challenges of tactile signals acquisition [129]. 

Prior to conducting any other signal processing task, it is essential to remove the unwanted signal disturbances by using artifact removal methods [132]. In practice, the acquired signals may be contaminated by different types of noise and distortions, such as instrumentation noise, powerline noise and other types of interference [133,134]. The suitability of specific artifact removal techniques greatly depends on the application and the nature of the sensor signals (e.g., data statistics, stationarity of the desired signal and the noise). A commonly-used artifact removal technique is bandpass filtering, implemented in the hardware or later in a digital software manner. However, filtering always fail to remove the artifacts when the desired signals and the artifacts overlap in the frequency domain or when the noise has a non-stationary nature [135]. To overcome this challenge, adaptive de-noising strategies, such as wavelet transform and empirical mode decomposition (EMD) are widely employed [133]. 

### 6.2. Smart Tactile Sensing Based on Machine Learning

Several works in the literature witness the adoption of machine learning algorithms for pattern-recognition tasks in tactile sensing systems [4,16,136,137,138,139,140,141,142,143,144,145,146,147,148]. The first stage of data mining typically is feature extraction. It provides a meaningful representation of the raw signals and can significantly reduce the amount of transmitted data through the tactile sensing system [4].

One common approach to represent tactile data is based on manually specified properties, such as the statistical characters of the time series signals, geometric properties of the object to touch [16]. For instance, Hoelscher et al. compared seven different methods of feature extraction from the preprocessed tactile signals [138], including physically motivated features, temporal BioTac features, Principal component analysis (PCA) of the raw data, mean features, pressure features, electrode features and temperature features. It was demonstrated that the robot could reliably classify 49 objects based on mean features from five robot motions [138]. 

In addition, a great deal of attention has been paid to extract features in unsupervised ways. Unsupervised feature learning can serve a wide range of applications and is also able to adapt to task-specific applications. For instance, Madry et al. proposed an unsupervised spatio-temporal feature learning method, named Spatio-Temporal Hierarchical Matching Pursuit (ST-HMP) [137,149]. The main idea is to extract features from the raw consecutive frames and then pool them over the time dimension. They further demonstrated the effectiveness of the proposed method on two tactile-based robotics applications, including the grasping stability assessment and object instance recognition [137]. Another powerful way to learn features without requiring prior knowledge is based on deep learning [144,149]. To extract features applicable in general tactile-based applications, Yuan et al. proposed a deep learning based method for shape-independent hardness estimation. They first represented frames of tactile imaging using a convolutional neural network (CNN) [150], and further adopted a recurrent neural network (RNN) [151] to model changes of the gel deformation over time [149]. As shown in [136,137], some of these handcrafted features might not be relevant to the given task. In addition, classification in high-dimensional feature spaces is prone to overfitting. Therefore, after feature extraction, it is always desirable to apply feature selection methods to select the best subset candidate features from the whole feature space [133].

**Figure 4:**
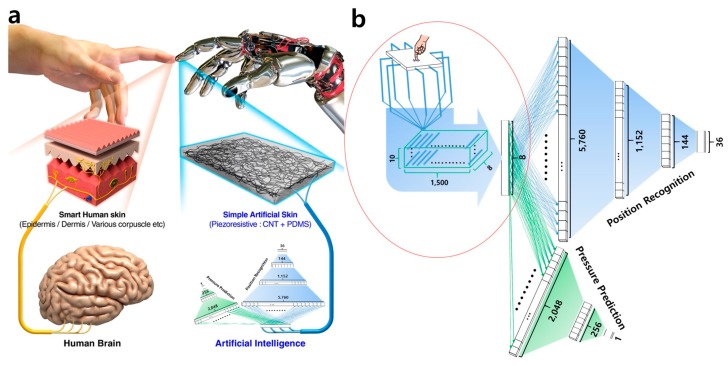
An illustrative study in [149] showing the basic concept for artificial skin and a DNN architecture for reliable sensing: (**a**) a schematic elucidating the comparison between the human skin and the artificial skin; and (**b**) the DNN architecture for tactile sensing. (Reprinted with permission from the authors [149] under the terms and conditions of the Creative Commons Attribution license (http://creativecommons.org/licenses/by/4.0/)).

Besides the quality of the acquired signals and the extracted/selected features, the performance of the smart tactile sensing system also depends on machine learning techniques. Each algorithm has its own advantages and limitations. For instance, the algorithm of K-Nearest Neighbors (KNN) is robust to noisy training data and effective when the training data is large. Naya et al. utilized the KNN principle to the haptic interface of a pet-like robot and aimed to recognize five touch modalities [145]. However, people need to determine the value of *K* and choose the suitable type of distance to get the best performance. In addition, the required time to find the nearest neighbors in a large training set can be excessive. Therefore, it is not suitable for real-time tactile sensing systems with limited computational ability. Support vector machines (SVMs) have also been successfully deployed in smart tactile sensing systems [146,147,148]. For instance, Gastaldo et al. utilized SVMs to tackle the interpretation of touch modalities in [146]. SVMs generally provide high accuracy, whereas they are memory intensive and are not recommended for small memory systems. Another important variety of machine learning methods is deep learning based methods. Recently, an extremely simple macroscale electronic skin without nano/micropatterns were realized with deep learning methods [149]. Figure 4 shows the Deep Neural Network (DNN) architecture used in this study. Deep learning enables the use of a simple bulky material for use in a smart sensory device (e.g., e-skin). The proposed revolutionary approach outperformed the present sensors in terms of pressure sensitivity and spatial resolution. The e-skin based on deep learning is unprecedented since all other currently available e-skins require a complicated and device-oriented construction and also depend on high-cost manufacturing processes [144]. Deep learning has been successful in multiple domains, outperforming traditional machine learning methods, if sufficient amount of training data is available [152,153]. The criterion to choose an appropriate method is subject to the nature of the acquired signals and the application of interest. Table 5 lists some existing smart tactile sensing systems and their utilized machine learning techniques.

### 6.3. Tactile Sensor Fusion

In reality, a human combines the sense of touch with other sensory modalities, such as vision and hearing, to form a coherent and robust perception of the world [15]. Tactile sensing systems relying on a single sensor could suffer from several limitations, such as limited spatial coverage and the system/data uncertainties. Therefore, the design of a smart tactile sensing system should take into account the presence of multiple sensors, for a signal sensory modality or multiple modalities. Similar to the human perceptual mechanism, a well-structured tactile sensing system collects information from the external environment by multiple sensors, such as the prosthetic limb equipped with more than 100 sensors [120]. The performance of a tactile sensing system is also highly determined by sensor data fusion algorithms. 

Generally, combining sensory information can yield better recognition performance. An effective way to combine multiple sensor data is represented as sensor fusion [133,155]. Sensor fusion can be performed at any stage of the signal processing and decision-making process, including the raw data-fusion, feature-fusion, and classifier-fusion.

Different sensor fusion methods can be adopted depending on the specific problem and the collected signals. If the sensors measure the same type of physical phenomena, the acquired signals from these sensors can be directly fused. Otherwise, data generated from heterogeneous sources may not be combined directly and it is generally preferred to be fused at the feature extraction stage or decision-making stage. As to feature-fusion, features extracted from multiple sensors separately can be concatenated into new high dimensional features as the input of the further classification/pattern recognition step. For instance, Jia et al. [156] used the sparse coding algorithm to extract features from three modalities independently, including the vibration, internal fluid pressure, and electrode impedance. The generated 200, 100 and 100 features from these sensor modalities are then fused via a fully connected layer. Classifier-fusion is the process of combining decisions generated from multiple “low level” classifiers. It attempts to get higher accuracy than those attainable from each individual classifier. Depending on the confidence of each classifier, decisions can be fused in a weighted voting manner. In [157], Halatci et al. proposed a classifier fusion method for planetary exploration rovers based on visual and tactile signals. It was demonstrated that more accurate terrain classification can be achieved via classifier fusion. For more related information, please refer to [15,155,157,158,159,160] and the references therein. 

## 7. Challenges for the Application of Tactile Sensing

Although developing smart tactile sensors has been an active area and has drawn increasing research attention in the last decades, the penetration of tactile sensors in commercial applications is still extremely low in comparisons with other sensing modalities. Today’s smart tactile sensing systems remain in their infancy. The remaining challenges include, but not limited to, the following:

(1) Cost. One of the challenges facing the researchers is finding a way to cut down the sophisticated tactile sensor system’s cost. Most existing tactile systems reported in the literature are still at the experiment level. It is desirable to get the cost down to a point affordable for the market.
(2) Hardware related to sensor performances (e.g., sensitivity, ability to measure various parameters), physical aspects (e.g., spatial resolution, conformability), tactile sensors arrangement, wireless communication and crosstalk. Nanotechnology and microfabrication may provide a way to integrate different sensing modalities and signal processing units. They further can provide a high density array of sensors.
(3) Software. Even if people have already developed numerous tactile sensors with fantastic characters, such as mimic the human sense of touch, tactile sensors are rarely used in real applications. Practical tactile sensing systems highly demand not only suitable hardware but also powerful software, especially for the systems working in unconstructed environments. The development of tactile sensing requires not only better sensors, but also efficient and effective techniques to process these sensors’ data. Difficulties with data acquisition and interpretation have consistently been cited as one main reason for the slow development.
(4) Modularization design and transportability. Ease of assembly and disassembly is another concern that should be better addressed. Tactile sensing systems, including the hardware and the software, are generally designed based on certain task-specific criteria. From the design point, modularization designs which can facilitate the transportability between different devices are highly desired.

## 8. Conclusions

In this paper, we overview fundamental factors to be concerned in the design and implementation of a smart tactile sensing system. As in the human perceptual mechanism, a smart sensing system not only needs to be equipped with a large number of receptors, tactile sensors, but also requires effective algorithms that can interpret the acquired information [3,4,125]. Therefore, the design issues of a smart tactile sensing system can be divided into two broad categories: physical aspects and software related issues. It is a multidisciplinary field requiring intensive interdisciplinary efforts and collaborations [2,15]. 

The performance of smart tactile sensing systems can be enhanced by exploring recent advances in the utilized materials and fabrication technologies, as well as signal processing and machine learning methods. Compared to some previous surveys of tactile sensing technologies, this review paper extends on previous reviews and emphasizes the state-of-the-art technologies to manufacture tactile sensors (especially factors related to sensor fabrication, structure and materials), outstanding signal processing methods which can effectively interpret the information from multiple sensors (numerous sensors for single modality or for multiple modalities), and challenges which must be overcome. Although the development of smart sensing systems remains in its infancy, the tactile sensing technology has great potential in enhancing people’s life quality. In this review, we mainly discuss the applications of smart tactile sensing technology in three fields: artificial skin for robotics, tactile sensing for unstructured environments, and biomedical applications.

## Figures and Tables

**Table 1:** Trade-offs of different sensing principles.

Sensing Principle	Trade-Offs	
	Sensing Structure Related	Read out System Related
Capacitive	High sensitivity and resolution	Highly integratable
	Large dynamic measurement range	Medium complexity
	Static and dynamic measurement	Medium power consumption
	Easily affected by noise	High portability
Piezo-resistive	High sensitivity and resolution	Highly integratable
	Robust to noise	Highly Low complexity
	In-situ structured sensor	High portability
	Susceptible to hysteresis	High power consumption
Piezoelectric	High sensitivity	Highly integratable
	Large dynamic range	Medium complexity
	High frequency response	Medium portability, little bulky
	Low spatial resolution	Medium power consumption
Optic	High sensitivity	Highly integrable
	Large dynamic range	Medium complexity
	High frequency response	Medium power consumption
	High spatial resolution	Medium portability

**Table 2:** Comparisons of tactile sensor material types.

Material Type	Patterning		Properties
	Deposit	Etch	
Silicic	High temperature	Highly dangerous chemical	Good mechanical properties
	High vacuum requirement		Tunable electrical conductivity
	Complex equipment	Complex equipment	Good thermal conductivity
	Low rate		Good optical properties
			High chemical stability
Metallic	Flexible temperature	Flexible and simpler etching method	Good electrical conductivity
	Flexible vacuum requirement		Good thermal conductivity
	Medium equipment complexity	Simpler equipment	Medium chemical stability
	Medium rate		
Polymer	Low temperature	Safe chemical	Medium to low mechanical properties
	Low vacuum requirement		Insulator
			High flexibility in functionalization
	Simple equipment	High rate	Good optical properties
	High rate		Low chemical stability, prone to oxidation

**Table 3:** Fabrication methods for different types of tactile sensors.

**Sensing Principles**	**Complexity and Cost**		
	**Surface/Bulk Machining**	**Mold/Imprinting**	**Rapid 3D Fabrication**
Capacitive	High	Medium	Low
Piezo-resistive	Medium	Medium	Low
Piezoelectric	Low	Medium	Low
Optic	Medium	Low	Low
**Sensing Principles**	**Robustness**		
	**Surface/Bulk Machining**	**Mold/Imprinting**	**Rapid 3D Fabrication**
Capacitive	Low	High	Low
Piezo-resistive	Medium	High	High
Piezoelectric	High	High	High
Optic	Medium	High	High

**Table 4:** Examples of existing tactile sensors for artificial skin.

Reference	Characters	Function
[104]	Pressure-sensitive, macroscale	Electronic skin capable of monitoring pressure with high spatial resolution
[106]	Energy-Autonomous, Flexible, and Transparent, sensitive to touch	Mimic human skin and can perform task ranging from simple touching to grabbing of soft objects
[107]	Ultra-lightweight, unbreakable and imperceptible	electronic skin, health care and monitoring and many others
[108]	Flexible, self-powered, self-clean	multi-functional e-skin, such as elbow bending or finger pressing
[109]	Unprecedented sensitivity for tactile pressure	Mimic human skin, with potential application in novel prosthetics and robotic surgery

**Table 5:** Some existing smart tactile sensing systems and the related machine learning techniques.

Reference	Tactile Sensors (Hardware)	Extracted Features	Machine Learning Method	Aim
[141]	BioTac (Pressure sensor)	Taction, roughness and fitness	Bayes	Texture classification
[143]	Tactile sensor array	226 features	Decision trees	Object identification
[137]	Schunk Dexterous, Schunk Parallel and iCub hands	Spatio-Temporal structures by unsupervised feature learning	Support vector machine (SVM)	Grasp stability assessment and object recognition
[149]	Macroscale electronic skin with a brilliant strain and position sensor	Features from electrical resistance change by DNN	Deep neural network (DNN)	Position recognition and pressure evaluation
[154]	GelSight Tactile Sensor	Features from tactile images by DNN	Deep convolutional and recurrent neural network	Hardness Estimation
[139]	Barometric pressure sensors	34 “haptic adjectives”	Random Forests	Estimation of metabolic equivalent of tasks
[145]	Humanoid robot, Cody, with force sensitive skin	Maximum force, contact area, and contact motion et al.	k-nearest neighbor (KNN)	Haptic classification and object recognition
[146]	A tactile sensing system with spatially distributed PVDF sensors	Spatial and temporal features from tactile imaging	Kernel-based Extreme Learning Machines and SVM	Interpretation of Touch modality
